# Kurtosis-Based Symbol Timing and Carrier Phase/Frequency Tracking

**DOI:** 10.3390/e23070819

**Published:** 2021-06-27

**Authors:** Todd K. Moon, Jacob H. Gunther

**Affiliations:** Electrical and Computer Engineering Department, Utah State University, Logan, UT 84322, USA; jake.gunther@usu.edu

**Keywords:** kurtosis, symbol timing estimation, carrier phase estimation, carrier frequency, offset estimation

## Abstract

Kurtosis is known to be effective at estimating signal timing and carrier phase offset when the processing is performed in a “burst mode,” that is, operating on a block of received signal in an offline fashion. In this paper, kurtosis-based estimation is extended to provide tracking of timing and carrier phase, and frequency offsets. The algorithm is compared with conventional PLL-type timing/phase estimation and shown to be superior in terms of speed of convergence, with comparable variance in the matched filter output symbols.

## 1. Introduction

In [1], a method was introduced for burst-mode symbol timing estimation and carrier phase estimation based upon complex and real kurtosis, respectively, of the received signal. The method involves computing kurtosis at several different parameter values (for both delay and phase) and is thus computationally expensive and more suited to offline computation than real-time implementations or parameter tracking. In this paper, kurtosis-based methods are extended to algorithms that track timing and phase. The carrier estimation is also extended to include both carrier phase and carrier frequency offset. As tracking algorithms (rather than burst-mode algorithms, which obtain one estimate for an entire burst), these algorithm are potentially amenable to real-time tracking application. In one development, tracking is accomplished by moving downhill on an objective function surface that operates without derivatives in a manner analogous to the Nelder–Mead simplex algorithm in one dimension [2]. In another development, a gradient descent algorithm is employed for phase/frequency estimation. The gradient descent method has higher complexity than the non-derivative method but has an otherwise similar performance. As shown in simulations, the kurtosis-based algorithms typically converge in fewer symbols than conventional PLL-type timing and phase tracking methods.

While [1] applied this kurtosis-based method only to QPSK constellations, in fact, it is agnostic with respect to signal constellation. Conventional synchronization algorithms typically employ knowledge of the signal constellation using training symbols and/or in a decision-directed mode (see, e.g., [3]). In some settings, such as in cognitive radio radio settings, in which an “intelligent receiver … adapt[s] itself to a specific transmission context and blindly estimate[s] the transmitter parameters for self-reconfiguration purposes” [4], signals with unknown signal constellations may be employed. It would be helpful to be able to perform symbol and carrier sync without knowledge of the constellation, following which constellation identification may be more readily undertaken. The algorithms presented here serve that purpose. In addition, many detection problems require the symbols to be appropriately scaled, which often requires the use of automatic gain control (AGC) loops as part of the synchronization process. It may be advantageous to decouple the AGC loop from symbol timing and phase estimation, which the kurtosis-based approach provides. The relatively fast convergence of the estimators may also make this useful in short blocklength scenarios for low latency communication systems.

The paper [1] did not contemplate the problem of residual carrier frequency offset, assuming instead that the Fourier transform-based approach to removing carrier offset is completely effective. In this paper, we account for residual carrier frequency offset.

Since kurtosis involves fourth powers of the data, outliers can have a significant effect that can lead an estimate astray or can result in higher variance of the estimated symbols. Another contribution of this paper is to introduce the use of a Huber function [5] to make the estimation more robust.

Kurtosis-based estimation has also been used for blind source separation [6,7,8], where the kurtosis is used as a measure of non-Gaussianity [9] (Section 8.2). Negentropy is also used as a measure of non-Gaussianity, where the negentropy of a random variable *y* is defined as [9] (Section 8.3)

$$J\left(y\right)=H\left(y_{\mathrm{gauss}}\right)-H\left(y\right)$$

where $y_{\mathrm{gauss}}$ is a Gaussian random variable with the same covariance as *y*. When the negentropy is approximated using higher-order cumulants, the negentropy can be expressed as

$$J\left(y\right)\approx \frac{1}{12}E{\left[y^{3}\right]}^{2}+\frac{1}{48}\mathrm{kurt}{\left(y\right)}^{2}.$$

For a zero-mean variable with symmetric distribution, the negentropy is thus essentially equivalent to the kurtosis. This relationship between an entropy-related quantity and the kurtosis is what suggested this article as a venue of publication.

Parameter estimation for communication systems has been widely studied. Textbooks on this topic include [10,11,12,13,14,15,16]. Parameter estimation for communication is also covered in conventional digital communication textbooks, e.g., [3,17,18]. See also [19]. What distinguishes this work from all of these references is the use of complex and real kurtosis as a primary tool for adaptation toward the parameter estimates, which enables the parameter estimators to operate agnostic of the signal constellation. While there are some methods that can be applied without knowing the transmitted signal, such as taking powers of the received data to remove digital symbol information, those methods work only with some constellations. By contrast, the kurtosis-based methods are more general. As shown below, they can converge quickly to parameter estimates, more quickly than, for example, PLL-based methods. This suggests the possibility of kurtosis-based phase estimation to be used in phase acquisition and, where the constellation is known, switching over to a PLL-type technique for tracking.

This paper focuses on single-carrier linearly modulated digital communication signals. Extension to multicarrier signals is a topic for future research.

## 2. Signal Model

Digital information is transmitted at a rate of $1/T_{s}$ symbols per second according to the complex bandpass representation

$$s\left(t\right)=\sum_{k}s_{k}p\left(t-kT_{s}\right)e^{j\omega_{0}t},$$

where p(t) is a unit-energy, pulse-shaping function satisfying the Nyquist zero ISI theorem (e.g., a square-root raised cosine, SRRC [3] (Appendix A)), $s_{k}=a_{k}+jb_{k}$ is a complex point from the signal constellation, and $\omega_{0}$ is the carrier frequency. The pulse p(t) is assumed to be symmetric so that the matched filter is the same as p(t). The received signal is

r(t)=s(t−τ)+n(t)

where τ is delay resulting from transmission through the channel and n(t) is noise, assumed to be 0 mean. At the receiver, the signal is bandpass filtered and basebanded using a frequency $\omega_{1}\approx \omega_{0}$. The resulting complex (nearly) basebanded signal is denoted as

$$u\left(t\right)=e^{-j\omega_{1}t}r\left(t\right)=\sum_{k}s_{k}p\left(t-kT_{s}-\tau \right)e^{j(\omega_{\mathrm{off}}t+\phi )}+n^{\prime }\left(t\right),$$

where $\omega_{\mathrm{off}}=\omega_{0}-\omega_{1}$ is the residual carrier frequency offset and ϕ accounts for the time delay and changes in index reference at the receiver.

This signal is rotated by an estimate of the offset frequency ${\hat{\omega }}_{\mathrm{off}}$ and passed through a matched filter with estimated delay $\hat{\tau }$ to produce the signal $x\left(t\right)=e^{-j{\hat{\omega }}_{\mathrm{off}}t}\left(u\left(t\right)*p\left(t-\hat{\tau }\right)\right)$, where * denotes convolution. The matched filter output can be expressed in terms of the pulse autocorrelation function

(1) $$x\left(t\right)=e^{j(\phi +\left(\omega_{\mathrm{off}}-{\hat{\omega }}_{\mathrm{off}}\right)t)}\sum_{k}s_{k}r_{p}\left(t-kT_{s}-\left(\tau -\hat{\tau }\right)\right)+z\left(t\right).$$

where z(t) represents the noise filtered through the matched filter and $r_{p}$ is the pulse autocorrelation function

$$r_{p}\left(t\right)=\int_{-\infty }^{\infty }p\left(\lambda \right)p\left(\lambda -t\right)\;d\lambda .$$

The representation in (1) is accurate provided that the frequency offset does not exceed about 5% of the symbol rate [16] restriction was pointed out by a reviewer).

In modern practice, of course, the processing steps described above are implemented in discrete time and filters must be truncated to finite length. The signal u(t) is sampled at a rate of *P* (an integer) samples per symbol. Using $T=T_{s}/P$, we write the basebanded sampled signal as $u\left[n\right]\overset{∆}{=}{\left.u(t)\right|}_{t=nT}$. The pulse-shaping function p(t) is truncated to finite duration, which we take to be (Q−1)T, where *Q* is an odd integer and *T* is the sampling interval. The pulse-shaping function is thus represented by *Q* samples, p(nT). Different authors employ different conventions for the pulse shaping function, representing it either as a noncausal signal, centered around 0, or as a causal signal. We use a notation that accommodates both convention. If the pulse p(t) is centered around t=0, then there are ⌊(Q−1)/2⌋ samples before and after n=0 and the peak of $r_{p}\left(nT\right)$ is at n=0. If p(t) and its matched filter are causal, then the samples of interest of p(t) occur at indices for n=0,1,…,Q−1. In this case, the peak of the autocorrelation function occurs at time t=(Q−1)T, corresponding to sample n=(Q−1). In either case, let *O* (“offset”) be the index offset at which the peak sample of $r_{p}$ occurs: O=0 for the pulse centered around the origin and O=Q−1 for the causal pulse.

Due to the zero-ISI property, samples of the autocorrelation at shifts of multiples of $T_{s}$ are 0:

$$r_{p}\left(OT+\ell T_{s}\right)=\left\{\begin{array}{cc} 1 & \ell =0\\ 0 & \ell \neq 0. \end{array}\right.$$

Let the downsampled signal be indexed so that the sample at n=0 corresponds to the full matched filter response of the first symbol. That is, for the causal pulse-shaping function and its matched filter, $x\left[n\right]={\left.x(t)\right|}_{t=(O+n)T}$ and $z\left[n\right]={\left.z(t)\right|}_{t=(O+n)T}$. This results in

$$x\left[n\right]=e^{j(\left(\omega_{\mathrm{off}}-{\hat{\omega }}_{\mathrm{off}}\right)nT+\phi )}\sum_{k}s_{k}r_{p}\left(OT+nT-kT_{s}-\left(\tau -\hat{\tau }\right)\right)+z\left[n\right].$$

Thus, x[0] corresponds to the symbol $s_{0}$, etc. In what follows, the noise terms z[n] is omitted for brevity. (The phase change due to the difference in *O* was absorbed here into the phase ϕ.)

The matched filter output is downsampled to the symbol rate, taking samples at indices n=ℓP, ℓ=0,1,…. The downsampled matched filter output is

$$\begin{array}{cc} y[\ell ] & =x[\ell P]\\ & =e^{j(\left(\omega_{\mathrm{off}}-{\hat{\omega }}_{\mathrm{off}}\right)\ell PT+\phi )}.\sum_{k}s_{k}r_{p}\left(OT+\left(\ell -k\right)T_{s}-\left(\tau -\hat{\tau }\right)\right) \end{array}$$

This can be decomposed into the term k=ℓ and the other terms are

(2) $$\begin{array}{cc} \; & y\left[\ell \right]=e^{j(\left(\omega_{\mathrm{off}}-{\hat{\omega }}_{\mathrm{off}}\right)\ell PT+\phi )}s_{\ell }r_{p}\left(OT-\left(\tau -\hat{\tau }\right)\right)+\\ \; & \;\underset{\mathrm{ISI}}{\underset{︸}{e^{j(\left(\omega_{\mathrm{off}}-{\hat{\omega }}_{\mathrm{off}}\right)kPT+\phi )}\sum_{k\neq \ell }s_{k}r_{p}\left(OT\;+\;\left(\ell \;-\;k\right)T_{s}\;-\;\left(\tau \;-\;\hat{\tau }\right)\right)}}. \end{array}$$

If $\hat{\tau }=\tau$, then the second sum, representing intersymbol interference (ISI), disappears and the downsampled matched filter output is $y\left[\ell \right]=s_{\ell }e^{j(\left(\omega_{\mathrm{off}}-{\hat{\omega }}_{\mathrm{off}}\right)\ell PT+\phi })$, a single rotated (and rotating) symbol output. On the other hand, when $\hat{\tau }\neq \tau$, the sample is corrupted by the terms in the ISI sum. The goal of the timing delay estimation problem is to determine $\hat{\tau }$ to eliminate the ISI. The goal of the phase/carrier offset problem is to eliminate the complex rotation factor $e^{j(\left(\omega_{\mathrm{off}}-{\hat{\omega }}_{\mathrm{off}}\right)\ell PT+\phi )}$.

## 3. Review of Kurtosis-Based Estimation

With this communication notation in place, we are now in a position to describe kurtosis-based parameter estimation. The kurtosis of a real zero mean random variable *y* is defined as [20,21]

(3) $$K_{r}\left(y\right)=E\left[y^{4}\right]-3E{\left[y^{2}\right]}^{2},$$

The kurtosis of a complex random variable is [21]

$$K_{c}\left(y\right)={E[|y|}^{4}{]-2E[|y|}^{2}{]}^{2}-{\left|E\left[y^{2}\right]\right|}^{2}.$$

The kurtosis (either real or complex) has the following key properties: (1) If *y* and *z* are independent random variables, then for constants *a* and *b*, $K\left(ay+bz\right)={\left|a\right|}^{4}K\left(y\right)+{\left|b\right|}^{4}K\left(z\right)$, and (2) the kurtosis of a Gaussian random variable is 0. For non-Gaussian random variables, the kurtosis may be greater than zero or less than zero. Random variables representing points drawn from a symbol constellation have negative kurtosis (that is, they are sub-Gaussian).

For a stationary (or nearly stationary) sequence of random variables $\left(y\left[1\right],y\left[2\right],\ldots ,y\left[N_{\mathrm{kurt}}\right]\right)$, the kurtosis may be estimated by using sample averages instead of expectations. Thus,

(4) $$\begin{array}{cc} {\hat{K}}_{c}\left(y\right) & =\frac{1}{N_{\mathrm{kurt}}}\sum_{n=1}^{N_{\mathrm{kurt}}}{\left|y\left(n\right)\right|}^{4}-2{\left[\frac{1}{N_{\mathrm{kurt}}}\sum_{n=1}^{N_{\mathrm{kurt}}}{\left|y\left(n\right)\right|}^{2}\right]}^{2}-\\ \; & \;\;{\left|\frac{1}{N_{\mathrm{kurt}}}\sum_{n=1}^{N_{\mathrm{kurt}}}y{\left(n\right)}^{2}\right|}^{2}, \end{array}$$

and, analogously, an estimate ${\hat{K}}_{r}$ is computed for the real kurtosis. Note that ${\hat{K}}_{c}\left(y\right)$ accepts an entire sequence of data as its argument, over which the averaging occurs to estimate the kurtosis.

**Figure 1:**
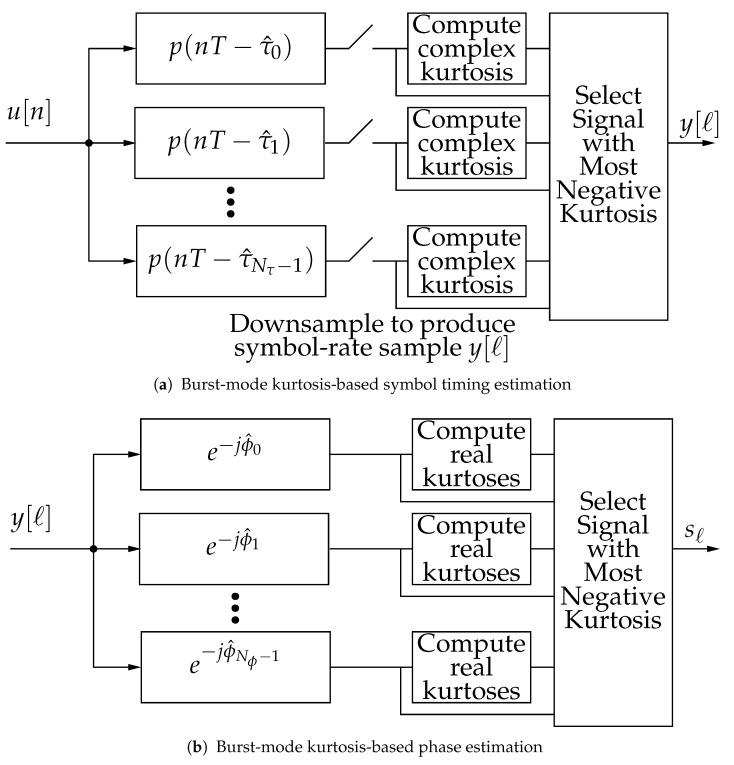
Block diagram of burst-mode kurtosis-based estimation.

The complex kurtosis of a sequence of matched filter outputs y[ℓ] may be used to determine $\hat{\tau }$ as follows. Considering the matched filter output in (2), when $\hat{\tau }\neq \tau$, by the central limit theorem, the sum of terms due to ISI causes y[ℓ] to tend toward having a Gaussian distribution, which has small absolute kurtosis. On the other hand, when $\hat{\tau }=\tau$, y[ℓ] consists only of the phase-rotated point from the signal constellation. Since the phase rotation does not affect the complex kurtosis, this y[ℓ] has negative kurtosis. This concept is used in [1] to synchronize a communication burst of many symbols (e.g., on the order of 100 symbols) using a method portrayed in Figure 1a. The received signal passes through a bank of $N_{\tau }$ matched filters, each having a different delay. (The burst-mode synchronization operation implies that the matched filtering is computed over the entire received sequence.) The delays $\tau_{0},\tau_{1},\ldots ,\tau_{N_{\tau }-1}$ uniformly sample the range $\left[0,T_{s}\right)$. The kurtosis of each of the downsampled matched filter outputs is computed, and the signal having the lowest (most negative) kurtosis is selected. That is,

$$\hat{\tau }=\arg\min_{\tau }\hat{K}c\left(u\left[n\right]*p\left(nT-\tau \right)\right)$$

Since complex magnitudes are employed in the complex kurtosis, a lack of knowledge about phase and carrier offset does not affect the estimation of the delay. By contrast, in conventional time and phase estimation (e.g., [3]), timing and phase are jointly estimated in a joint computational structure, so the convergence of one estimate affects the convergence of the other.

The paper [1] recommends determining carrier frequency offset $\omega_{\mathrm{off}}$ using Fourier transform techniques. This is still recommended when using the methods described in this paper when the carrier frequency offset exceeds the ability of the algorithm to track. However, even after removing the carrier frequency offset using Fourier transform techniques, some residual carrier may remain to be tracked, which we still refer to as $\omega_{\mathrm{off}}$. The method presented below reliably perform such offset frequency tracking.

For the moment, however, assume that the frequency offset is removed, so that, when the symbol timing is correctly estimated, the matched filter output is $y\left[\ell \right]=s_{\ell }e^{j\phi }$. In terms of real and imaginary parts, $y\left[\ell \right]=y_{r}\left[\ell \right]+jy_{i}\left[\ell \right]$ and $s_{\ell }=a_{\ell }+jb_{\ell }$. By stacking these components, the rotation can be written, and using

$${}_{\ell }=\left[\begin{array}{c} a_{\ell }\\ b_{\ell } \end{array}\right]\;G\left(\phi \right)=\left[\begin{array}{cc} c(\phi ) & -s(\phi )\\ s(\phi ) & c(\phi ) \end{array}\right]\;\mathrm{and}\;\begin{array}{c} c(\phi )=\cos(\phi )\\ s(\phi )=\sin(\phi ), \end{array}$$

the rotated symbols can be written as

(5) $$y_{\ell }\overset{∆}{=}\left[\begin{array}{c} y_{r}\left[\ell \right]\\ y_{i}\left[\ell \right] \end{array}\right]=G{\left(\phi \right)}_{\ell }=\left[\begin{array}{c} c\left(\phi \right)a_{\ell }-s\left(\phi \right)b_{\ell }\\ s\left(\phi \right)a_{\ell }+c\left(\phi \right)b_{\ell } \end{array}\right].$$

According to (5), the rotation G(ϕ) produces a linear combination of the real and imaginary components of the symbol. Computing the kurtosis (separately) on the real and imaginary components $y_{r}\left[\ell \right]$ and $y_{i}\left[\ell \right]$, the kurtosis is smaller (nearer to zero) when ϕ≠0 due to the mixture of $a_{\ell }$ and $b_{\ell }$. Remarkably, even though $y_{r}$ and $y_{i}$ are mixtures of only two random variables, the presence of the mixture can be detected using kurtosis.

A phase estimate $\hat{\phi }$ is selected and minimizes the sum of the kurtosis of the real and imaginary parts:

$$\hat{\phi }=\arg\min_{\phi }\left({\hat{K}}_{r}\left(y_{r}\right)+{\hat{K}}_{r}\left(y_{i}\right)\right)$$

The phase offset can be removed by computing the product $y_{\mathrm{rot}}\left[\ell \right]=e^{-j}y\left[\ell \right]$. This can be expressed in matrix/vector form by stacking real and imaginary parts, leading to

$$y_{\ell ,\mathrm{rot}}=G(-\hat{\phi })y_{\ell }=G\left(\phi -\hat{\phi }\right)s_{\ell }.$$

When $\hat{\phi }=\phi$, the components of $y_{\ell ,\mathrm{rot}}$ are not mixed, so that the sum of the real kurtosis of the real and imaginary parts are maximally negative.

This kurtosis-based estimation is applied as shown in Figure 1b. There are $N_{\phi }$ different rotations which span [0,2π). The time-synchronized matched filter output y[ℓ] is rotated by each rotation, and the sum of the kurtosis of the real part and the kurtosis of the imaginary part are computed. The symbol having is determined from the kurtosis that is smallest (most negative) kurtosis.

## 4. Symbol Timing Tracking: Discrete Downhill Minimization

The kurtosis-based methods portrayed in Figure 1, representing the method in [1], require evaluation using $N_{\tau }$ different matched filters in the symbol timing sync, followed by the same number of kurtosis computations, or $N_{\phi }$ phase rotations, followed by the same number of kurtosis computations, where the kurtosis was computed using data over an entire burst. In [1], the expense of these computations is ameliorated by the fact that these computations are performed only once per burst. However, there is no provision in [1] to re-estimate or track the parameters. For this reason, the method is referred to as a “burst mode” algorithm, suitable for one-time estimation on a burst, without adaptation. The present paper computes only two kurtosis values for each symbol and computes the kurtosis over smaller segments of the signal, which reduces computational complexity (for each kurtosis). The authors found it surprising that, even using rather short segments to estimate the kurtosis (resulting in noisy estimates of the kurtosis), these kurtosis estimates were able to be used to estimate the timing and phase parameters.

This section describes how to use two kurtosis values computed for each symbol using a method that “slides downhill” toward the most negative kurtosis to estimate the timing offset. Only two kurtosis values are computed per symbol (as opposed to evaluating at $N_{\tau }$ different values). This adaptive downhill slide is able to track changes in the parameters.

Let the (ostensibly) basebanded signal be denoted as u[n]. It is assumed that it is available over the necessary length of indices (such as by buffering).

Let the sampled $N_{\tau }$ delayed matched filters be denoted as follows:

(6) $$p_{i}\left[n\right]=p\left(nT-\tau_{i}\right),\;n=0,1,\ldots ,Q-1,i=0,1,\ldots ,N_{\tau }-1.$$

The index *i* must be in the range $0,1,\ldots ,N_{\tau }-1$. To ensure that is the case, we use the notation $p_{i\%N_{\tau }}\left[n\right]$, where $i\%N_{\tau }$ denotes *i* mod $N_{\tau }$. In (6), the range of *n* is for causal pulses, in which case, *O* is Q−1, as discussed above. For 0-centered pulses, n=⌊(Q−1)/2⌋,…,0,…,⌊(Q−1)/2⌋, and O=0.

In this paper, instead of evaluating kurtosis at $N_{\tau }$ different timing offsets, at each symbol time, kurtosis is evaluated at two timing estimates. Let $i_{1}$ and $i_{2}$ be the indices of delay of the matched filters being used at the present time, where the indices refer to the delay estimates $\tau_{i_{1}}$ and $\tau_{i_{2}}$. In order to produce the downsampled matched filter outputs used to estimate kurtosis, $L=Q+(N_{\mathrm{kurt}}-1)P$ input symbols are convolved with the matched filters $p_{i_{1}}$ and $p_{i_{2}}$, resulting in the matched filter outputs

$$x_{m}\left[n\right]=u\left[n:N+L-1\right]*p_{i_{m}\%N_{\tau }}\left[n\right],\;m=1,2,$$

where * denotes convolution. The downsampled matched filter samples are indexed so that the first retained matched filter sample is at n=0 are

$$y_{m}=x_{m}\left[O:P:O+\left(N_{\mathrm{kurt}}-1\right)P\right],\;m=1,2.$$

Let

$$K_{m}=\mathrm{complexkurtosis}\left(y_{m}\right),\;m=1,2,$$

denote the complex kurtosis estimated from the sequence $y_{m}\left[n\right]$, computed as in (4).

The minimization algorithm seeks to minimize the kurtosis $K_{m}$ over a series of symbol times. The minimization operates analogous to a one-dimensional Nelder–Mead algorithm [2], rolling downhill toward minimum kurtosis by moving in the direction of smaller kurtosis. This is referred to as discrete downhill minimization. It has been found by experimentation on communications data that the kurtosis is, in fact, a convex function of the delay.

**Figure 2:**
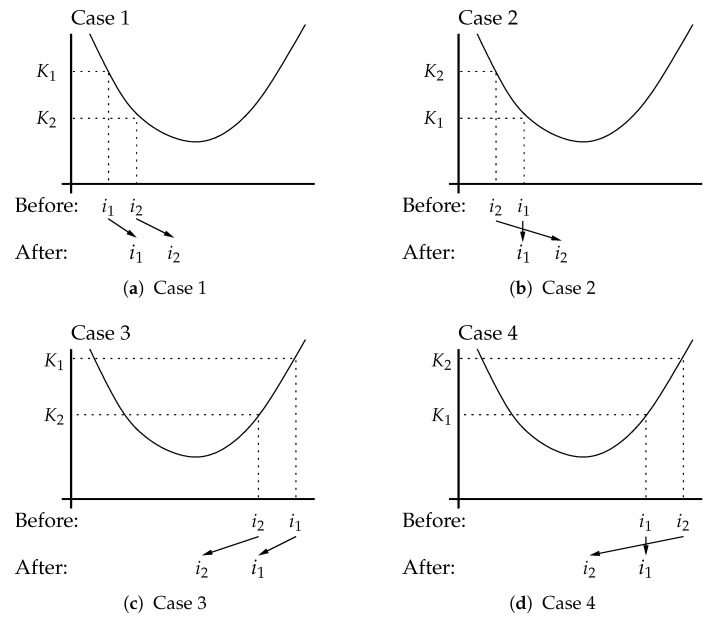
Discrete downhill minimization of kurtosis function.

At some symbol time of the two kurtoses $K_{1}$ and $K_{2}$, computed using $p_{i_{1}}$ and $p_{i_{2}}$, the lower (closer to −∞) kurtosis is retained, the downsampled matched filter output is saved in $y_{\mathrm{best}}$, and the index of the other delay is adjusted by $n_{\tau }$ steps to attempt to move toward lower kurtosis. The operation is detailed in Figure 2 and the logic is explicitly described in lines 48–60 of Algorithm 1 below. After execution of the steps, $i_{1}$ indexes the delay with lower kurtosis and $i_{2}$ is ready to be tested at the next time step. For example, in Case 1, since $K_{1}>K_{2}$, $i_{1}$ is set to the value $i_{2}$ and $i_{2}$ is adjust by some number of steps $n_{\tau }$. $n_{\tau }$ is a small integer, say 2 or 3, indicating how fast to adapt. The other cases in Figure 2 correspond to other configurations of the kurtosis as a function of the delay indices $i_{1}$ and $i_{2}$.

If $i_{1}$ already indexes the delay of minimum kurtosis, $i_{2}$ bounces back and forth between $i_{1}-n_{\tau }$ and $i_{1}+n_{\tau }$, leaving $i_{1}$ at the delay $\tau_{i_{1}}$, producing the lowest kurtosis. The descent algorithm moves at $n_{\tau }$ at each step, so that the average number of steps to converge is $N_{\tau }/\left(2n_{\tau }\right)$.

The sequence of matched filter outputs selected with the lowest kurtosis is referred to as $y_{\mathrm{best}}$.

The number of steps of adjustment, $n_{\tau }$, is adjusted to provide a variable stepsize algorithm. The variance in the changes in index values is computed. When the variance is below a threshold (suggesting that the estimate is converging to a steady value) $n_{\tau }$ is decremented, provided that it is >1. This reduces the jitter of the estimate in steady state. This dynamic encourages rapid convergence. Tuning of the algorithm can be performed by adjusting the decision thresholds. (While not totally satisfying, this is not so different from a PLL-based estimator, for which tuning may be required to obtain a desired performance.)

When an index falls outside the range $0,1,\ldots ,N_{\tau }-1$, it should be reduced modulo $N_{\tau }$. However, in order to preserve the directional ordering between $i_{1}$ and $i_{2}$, this modulo reduction occurs only when both indices fall outside this range. Then both indices are reduced. These operations are described in lines 69–77 of Algorithm 1.

## 5. Carrier Frequency and Phase Tracking: Discrete Downhill Minimization

In this section, a minimization technique is developed for carrier frequency and phase tracking, similar to that used in the previous section for timing synchronization. This allows the algorithm to track these parameters.

The phase is estimated to have one of $N_{\phi }$ different values $\phi_{0},\phi_{1},\ldots ,\phi_{N_{\phi }}$, which uniformly sample the range [0,2π). Let $\Delta_{\phi }$ denote the increment in phase between adjacent steps, e.g., $\Delta_{\phi }=\phi_{1}-\phi_{0}$.

Phase is estimated by rotating $y_{\mathrm{best}}$ using two values of phase indexed by $j_{1}$ and $j_{2}$ and an estimate of $\omega_{\mathrm{off}}$, then by estimating the real kurtosis on the real and imaginary parts of the signal, and by using this information to move downhill. The rotating signals are produced by

$$y_{\phi 1}=e^{-j(\hat{\omega }(0:N_{\mathrm{kurt}\phi }-1)+\phi_{j_{1}\%N_{\phi }})}⊙y_{\mathrm{best}}$$

$$y_{\phi 2}=e^{-j(\hat{\omega }\left(0:N_{\mathrm{kurt}\phi }-1\right)+\phi_{j_{2}\%N_{\phi }})}⊙y_{\mathrm{best}},$$

where ⊙ denotes element-by-element multiplication. The real kurtosis is computed as

$$J_{1}=\mathrm{realkurtosis}\left(\left(y_{\phi 1}\right)\right)+\mathrm{realkurtosis}\left(\left(y_{\phi 2}\right)\right)$$

and similarly for $J_{2}$.

The frequency is estimated as follows. Let $j_{\mathrm{last}}$ denote the phase index of the best phase at the previous time. The change in phase from the last to the current time is $\Delta \phi =\left(\delta_{\phi }\right)\left(j_{1}-j_{\mathrm{last}}\right)$. With an index changes of 0 or ±1, Δϕ may be thought of as a discrete-time point process taking values 0 and ±δϕ. The average value of this point process is the frequency estimate $\hat{\omega }$. The frequency estimate $\hat{\omega }$ taken as the output of a single-pole lowpass filter with unit DC gain and with input Δϕ. The pole of the filter is denoted by α. The filtering is computed according to $\hat{\omega }=\Delta \left(1-\alpha \right)+\alpha {\hat{\omega }}_{\mathrm{last}}$, ${\hat{\omega }}_{\mathrm{last}}=\hat{\omega }$.

The following pseudocode (Algorithm 1) summarizes the timing and phase estimation algorithms.    
**Algorithm 1:** Kurtosis-based timing and phase tracking1 function [mfout,cumwraptime ] = tracker(*u*,*n*) 2 **Internal (persistent or class) data**: 3    P= number of samples per symbol (integer) 4    $N_{\mathrm{kurt}}=$ number of symbols used to estimate kurtosis 5    Q= number of samples in each SRRC pulse 6    $L=Q+(N_{\mathrm{kurt}}-1)P=$ number of samples used in convolution 7   Variables for time estimation 8    $N_{\tau }=$ number of delay steps to consider 9    $\tau_{i}=$ array of $N_{\tau }$ delays in the range $\left[0,T_{s}\right)$
10    $i_{1},i_{2}$ = indices into $\tau_{i}$ array **0-based indexing is used. Init: $i_{1}=0$, $i_{2}=\left\lfloor N_{\tau }/2\right\rfloor$**
11   Stored values of the delayed SRRC pulse $p_{i}\left[n\right]\overset{∆}{=}p\left(nT-\tau_{i}\right)$,               for n=(0,1,…,Q−1) and $i=(0,1,\ldots ,N_{\tau }-1)$
12   cumwraptime = cumulative wrap count. Init = 0 13   Variables for variable stepsize algorithm 14   $n_{\tau }$ = number of steps. Init = 2 or 3 15   $L_{\mathrm{hist}}$ = length of $\tau_{1}$ history (a circular buffer) 16   hist${}_{\tau }$ = buffer history of $i_{1}$ (length = $L_{\mathrm{hist}}$) 17   $k_{\tau }$ = index into ${\mathrm{hist}}_{\tau }$. Init = 0 18   $N_{\mathrm{hist}}$ = number of elements in histτ. Init = 0 19   $\sigma_{\tau }$ = variance of elements in hist${}_{\tau }$
20   ${\mathrm{thresh}}_{\tau }$ = variance threshold to reduce stepsize 21   Variables for phase/frequency estimation 22   $N_{\phi }$ number of phase steps to consider 23   $\phi_{j}=$ array of $N_{\phi }$ phases in [0,2π)
24   $\delta \phi =2\pi /(N_{\phi }-1)$ = phase step size 25   ϕstepsize = number of index steps to move in phase adaptation. Init = 1 26   $j_{1},j_{2}$ = indices into $\phi_{j}$ array. Init: $j_{1}=0$, $j_{2}=\left\lfloor N_{\phi }/2\right\rfloor$
27   lastj = last value of $j_{1}$ or $j_{2}$. Init=0 28   cumwraphphase = cumulative phase wrap count. Init = 0 29   Variables for frequency estimation 30    $\hat{\omega }$ = frequency offset estimate. Init = 0 31   lastomega = previous value of $\hat{\omega }$. Init = 0 32   α = pole location of single-pole LPF. α≈0.95
33 **Inputs:**
34   *u* = received basebanded signal (function uses *L* samples in u[n:n+L−1]) 35   *n* = starting sample of s[n] at this iteration (*n* increased by *P* before each call) 36 **Output:**
37   mfout = synched/rotated matched filter output 38   cumwraptime = total number of timing wraparounds 39   (**Timing estimation**) 40    $x_{1}$ = $u\left[n:N+L-1\right]*p_{i_{1}\%N_{\tau }}$    (MF outputs (* = convolution)) 41   $x_{2}$ = $u\left[n:N+L-1\right]*p_{i_{2}\%N_{\tau }}$ 42   $y_{1}=x_{1}\left[Q\;-\;1:P:Q\;-\;1\;+\;L\right]$ 43   (downsampled ($N_{\mathrm{kurt}}$ symbols out)) 44   $y_{2}=x_{2}\left[Q\;-\;1:P:Q\;-\;1\;+\;L\right]$ 45   $K_{1}=$complexkurt($y_{1}$)    (Compute kurtosis using (4)) 46   $K_{2}=$complexkurt($y_{2}$) 47   (Move downhill toward best delay:) 48   if $K_{2}<K_{1}$ and $i_{1}<i_{2}$    (Case 1) 49      $i_{1}=i_{2}$; $K_{1}=K_{2}$; $i_{2}$ += $n_{\tau }$ 50      $y_{\mathrm{best}}=y_{2}$    (assign the entire sequence) 51   elseif $K_{1}\leq K_{2}$ and $i_{2}<i_{1}$    (Case 2) 52      $i_{2}=i_{1}+n_{\tau }$ 53      $y_{\mathrm{best}}=y_{1}$ 54   elseif $K_{2}<K_{1}$ and $i_{2}<i_{1}$    (Case 3) 55      $i_{1}=i_{2}$; $i_{2}$−= $n_{\tau }$; $K_{1}=K_{2}$ 56      $y_{\mathrm{best}}=y_{2}$ 57   elseif $K_{1}<K_{2}$ and $i_{1}<i_{2}$    (Case 4) 58      $i_{2}=i_{1}-n_{\tau }$ 59      $y_{\mathrm{best}}=y_{1}$ 60   end 61   (Adjust the step size) 62   tauhist$\left[k_{\tau }\right]=i_{1}$ 63   $k_{\tau }=\left(k_{\tau }+1\right)\%L_{\mathrm{hist}}$ 64   $N_{\mathrm{hist}}$ = min($N_{\mathrm{hist}}$+1, $L_{\mathrm{hist}}$ ) 65   if($N_{\mathrm{hist}}$ == $L_{\mathrm{hist}}$) 66      $\sigma_{\tau }$ = (tauhist) 67      if($\sigma_{\tau }<{\mathrm{thresh}}_{\tau }$ and $n_{\tau }>2$) $n_{\tau }$−= 1 68   end 69    (If both delays $\geq N_{\tau }$ or both <0, wrap around:) 70    wrap = 0 71    if $i_{1}\geq N_{\tau }$ and $i_{2}\geq N_{\tau }$ 72       $i_{1}-$= $N_{\tau }$; $i_{2}-$= $N_{\tau }$ 73       wrap = 1 74    elseif $i_{1}<0$ and $i_{2}<0$ 75       $i_{1}$ += $N_{\tau }$; $i_{2}$ += $N_{\tau }$ 76       wrap = −1 77    end 78   (At this point, $y_{\mathrm{best}}$ is an array containing 79   $N_{\mathrm{kurt}}$ time-aligned MF outputs) 80   **(Carrier offset/frequency estimation)** 81   despin1 = $e^{-j(\phi_{j_{1}\%N_{\phi }}+\hat{\omega }\left(0:N_{\mathrm{kurt}}-1\right))}$    (array with $N_{\mathrm{kurt}}$ elements) 82   despin2 = $e^{-j(\phi_{j_{2}\%N_{\phi }}+\hat{\omega }\left(0:N_{\mathrm{kurt}}-1\right))}$ 83   $y_{\phi 1}=y_{\mathrm{best}}⊙\mathrm{despin}1$    (De-rotate the matched filter outputs) 84   $y_{\phi 2}=y_{\mathrm{best}}⊙\mathrm{despin}2$ 85   $J_{1}=\mathrm{realcomplexkurt}\left(y_{\phi 1}\right)$    (∑ kurtosis on real & imag) 86   $J_{2}=\mathrm{realcomplexkurt}\left(y_{\phi 2}\right)$ 87   (Move downhill toward best phase:) 88   if $J_{2}\leq J_{1}$ and $j_{1}<j_{2}$ 89      $j_{1}=j_{2}$; $K_{1}\;K_{2}$; $j_{2}$+= ϕstepsize 90      $\mathrm{mfout}=y_{\phi 2}\left[0\right]$    (save matched filter output) 91   elseif $J_{1}\leq J_{2}$ and $j_{2}<j_{1}$ 92      $j_{2}=j_{1}+\phi \mathrm{stepsize}$ 93      $\mathrm{mfout}=y_{\phi 1}\left[0\right]$ 94   elseif $J_{2}\leq J_{1}$ and $j_{2}<j_{1}$ 95      $j_{1}=j_{2}$; $K_{1}=K_{2}$ 96      $j_{2}-$= ϕstepsize 97      $\mathrm{mfout}=y_{\phi 2}\left[0\right]$ 98   elseif $K_{1}<K_{2}$ and $j_{1}<j_{2}$ 99      $j_{2}$−= ϕstepsize 100      $\mathrm{mfout}=y_{\phi 1}\left[0\right]$ 101   end 102   $\Delta j=j_{1}-\mathrm{last}j$;    (phase difference in counts) 103   Δϕ=(Δj)(δϕ)    (phase difference in rads) 104   wrap = 0    (Do phase wrap around) 105   if $j_{1}\geq N_{\phi }$ and $j_{2}\geq N_{\phi }$ 106       $j_{1}$−= $N_{\phi }$; $j_{2}$−= $N_{\phi }$; 107        wrap=−1; 108   elseif $j_{1}<0$ and $j_{2}<0$ 109       $j_{2}$+= $N_{\phi }$; $j_{2}$+= $N_{\phi }$; 110        
wrap=1 111   end 112   $\mathrm{last}j=j_{1}$ 113   cumwrapphase+=wrap 114   Estimate the frequency by smoothing Δϕ 115   
$\hat{\omega }=\Delta \phi (1-\alpha )+\alpha \;\mathrm{lastomega}$ 116   
$\mathrm{lastomega}=\hat{\omega }$ 117   Adjust the phase adjustment step size 118   
$\phi \mathrm{stepsize}=\lfloor |\hat{\omega }|/(\delta \phi )\rfloor +1$ 119 end function

## 6. Huber Loss Function

The fourth moments computed in the kurtosis open the algorithm to the vulnerability that outlier events unduly affect the estimate. This effect can be mitigated by employing a Huber loss function [5], which is commonly used for robust estimation. This function is defined as follows:

$$H_{\delta_{H}}\left(a\right)=\left\{\begin{array}{cc} \frac{1}{2}a^{2} & \mathrm{if}\;|a|\leq \delta_{H},\\ \delta_{H}\left(|a\right|-\frac{1}{2}\delta_{H}) & \mathrm{otherwise}. \end{array}\right.$$

$H_{\delta_{H}}\left(a\right)$ behaves quadratically for small values of *a* (when $\left|a\right|\leq \delta_{H}$) and linearly for larger values of *a*, with the transition defined such that the transition from quadratic behavior to linear behavior at $\left|a\right|=\delta_{H}$ is continuous and continuously differentiable. The real kurtosis of (3) is approximated using the Huber function by

$$K_{r}^{H}\left(y\right)=E\left[H_{\delta_{H}}\left(H_{\delta_{H}}\left(y\right)\right)\right]-3H_{\delta_{H}}\left(E\left[H_{\delta_{H}}\left(y\right)\right]\right)$$

In complex kurtosis, for the terms involving the magnitude |y|, the Huber function can be applied directly. For the term involving $y^{2}$, the Huber function is applied to the magnitude, leaving the phase quadratically varying. The complex Kurtosis is approximated using the Huber function as follows:

$$\begin{array}{cc} K_{c}^{H}\left(y\right) & =E[H_{\delta_{H}}(H_{\delta_{H}}\left(|y|))\right]-2H_{\delta_{H}}(E[H_{\delta_{H}}\left(|y|)]\right)-\\ & \;H_{\delta_{H}}\left(\left|E[H_{\delta_{H}}\left(|y|\right)e^{j2∠y}]\right|\right). \end{array}$$

This is estimated from a sequence of observations:

$$\begin{array}{cc} {\hat{K}}_{c}^{H}\left(y\right) & =\frac{1}{N_{\mathrm{kurt}}}\sum_{n=1}^{N_{\mathrm{kurt}}}H_{\delta_{H}}(H_{\delta_{H}}\left(|y\left(n\right)|)\right)-\\ & \;2H_{\delta_{H}}\left(\frac{1}{N_{\mathrm{kurt}}}\sum_{n=1}^{N_{\mathrm{kurt}}}H_{\delta_{H}}\left(|y\left(n\right)|\right)\right)-\\ & \;H_{\delta_{H}}\left(\left|\frac{1}{N_{\mathrm{kurt}}}\sum_{n=1}^{N_{\mathrm{kurt}}}H_{\delta_{H}}\left(|y\left(n\right)|\right)e^{j2∠y(n)}\right|\right). \end{array}$$

Huber functions can be used in Algorithm 1 by simply replacing the computations of $K_{1}$ and $K_{2}$ at lines 45 and 46 with the complex Huber function, and the computations of $J_{1}$ and $J_{2}$ at lines 85 and 86 with the real Huber function.

## 7. Gradient Descent for Phase Estimation

As an alternative to optimizing over a fixed number of alternatives, gradient descent may also be employed. We illustrate the method here for phase estimation; this can be modified for timing estimation.

Let the real and imaginary components of the rotated signal be denoted as

$$\left[\begin{array}{c} y_{r,rot}\\ y_{i,rot} \end{array}\right]=\left[\begin{array}{c} c\left(\hat{\phi }\right)y_{r}+s\left(\hat{\phi }\right)y_{i}\\ -s\left(\hat{\phi }\right)y_{r}+c\left(\hat{\phi }\right)y_{i} \end{array}\right]$$

where $c(\hat{\phi })=\cos(\hat{\phi })$ and $s(\hat{\phi })=\sin(\hat{\phi })$. The objective function is the sum of the real kurtoses of the real and imaginary parts. Expanding this and identifying the moment terms using the variables *A* through *D*, we find

$$\begin{array}{cc} & J\left(\hat{\phi }\right)=K_{r}\left(y_{r,rot}\right)+K_{r}\left(y_{i,rot}\right)\\ & \;=E\left[{\left(c\left(\hat{\phi }\right)y_{r}+s\left(\hat{\phi }\right)y_{i}\right)}^{2}\right]-3(E{\left[{\left(c\left(\hat{\phi }\right)y_{r}+s\left(\hat{\phi }\right)y_{i}\right)}^{2}\right]}^{2}+\\ & \;\;E\left[{\left(-s\left(\hat{\phi }\right)y_{r}+c\left(\hat{\phi }\right)y_{i}\right)}^{2}\right]-3(E{\left[{\left(-s\left(\hat{\phi }\right)y_{r}+c\left(\hat{\phi }\right)y_{i}\right)}^{2}\right]}^{2}\\ & \;=c{\left(\hat{\phi }\right)}^{4}\underset{A}{\underset{︸}{\left(E\left[y_{r}^{4}\right]-3E{\left[y_{r}^{2}\right]}^{2}+4E\left[y_{i}^{4}\right]-3E{\left[y_{i}^{2}\right]}^{2}\right)}}\\ & \;+c{\left(\hat{\phi }\right)}^{3}s\left(\hat{\phi }\right)\times \\ & \underset{B}{\underset{︸}{\left(4E\left[y_{r}^{3}y_{i}\right]-4E\left[y_{r}y_{i}^{3}\right]-12E\left[y_{r}^{2}\right]E\left[y_{r}y_{i}\right]+12E\left[y_{i}^{2}\right]E\left[y_{r}y_{i}\right]\right)}}\\ & \;+c{\left(\hat{\phi }\right)}^{2}s{\left(\hat{\phi }\right)}^{2}\underset{C}{\underset{︸}{\left(12E\left[y_{r}^{2}y_{i}^{2}\right]-12E\left[y_{r}^{2}\right]E\left[y_{i}^{2}\right]\right)}}\\ & \;+c\left(\hat{\phi }\right)s{\left(\hat{\phi }\right)}^{3}\times \\ & \;\underset{D}{\underset{︸}{\left(4E\left[y_{r}y_{i}^{3}\right]-12E\left[y_{r}y_{i}\right]E\left[y_{i}^{2}\right]-4E\left[y_{r}^{3}y_{i}\right]+12E\left[y_{r}^{2}\right]E\left[y_{r}y_{i}\right]\right)}}\\ & \;+s{\left(\hat{\phi }\right)}^{4}\underset{A}{\underset{︸}{\left(E\left[y_{i}^{4}\right]+E\left[y_{r}^{4}\right]-3E{\left[y_{i}^{2}\right]}^{2}-3E\left[y_{r}^{2}\right]E\left[y_{i}^{2}\right]\right)}} \end{array}$$

The gradient of $J(\hat{\phi })$ is

$$\begin{array}{cc} & J(\hat{\phi })=A(4s{\left(\hat{\phi }\right)}^{3}c\left(\hat{\phi }\right)-4c{\left(\hat{\phi }\right)}^{3}s\left(\hat{\phi }\right))\\ & \;+B(c{\left(\hat{\phi }\right)}^{4}-3s{\left(\hat{\phi }\right)}^{2}c{\left(\hat{\phi }\right)}^{2})+\\ & C\left(-2c\left(\hat{\phi }\right)s{\left(\hat{\phi }\right)}^{3}+2c{\left(\hat{\phi }\right)}^{3}s\left(\hat{\phi }\right)\right)+D\left(3c{\left(\hat{\phi }\right)}^{2}s{\left(\hat{\phi }\right)}^{2}-s{\left(\hat{\phi }\right)}^{4}\right) \end{array}$$

## 8. Experimental Results

Kurtosis-based estimation was compared with the PLL-based timing and phase synch algorithms of [3] (Sections 7.4 and 8.4.4). In these algorithms, the timing interpolator is governed by the fractional symbol μ. The phase estimate is represented by the DDS (direct digital synthesizer) value. Two examples are presented here, one with $\omega_{\mathrm{off}}=0$ and one with $\omega_{\mathrm{off}}=0.01$.

**Example** **1.** *QPSK, SNR = 10 dB, excess bandwidth = 0.2. $\omega_{off}=0$. Algorithm parameters: $N_{kurt}=20$, $N_{\tau }=60$, $N_{\phi }=40$, Q=101. ω-filtering parameter α=0.99.*

**Figure 3:**
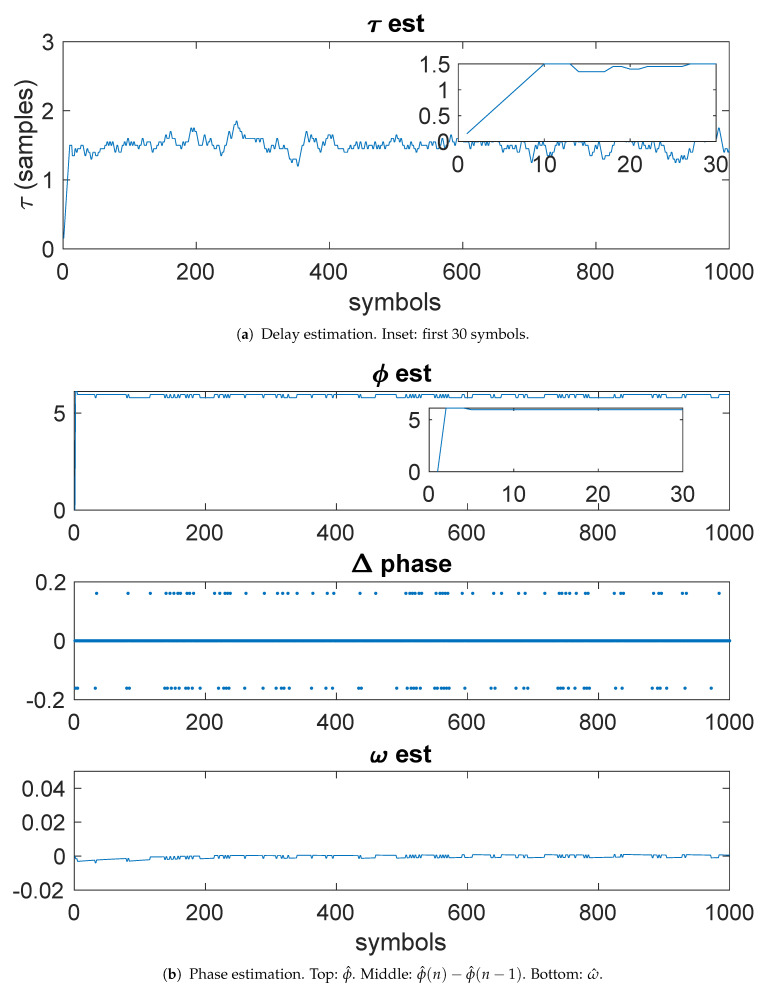
Example 1: Kurtosis-based timing and phase estimation.

Figure 3a shows the results of the delay estimation. The inset in the figure shows the first 30 samples, illustrating that convergence has occurred in about 10 symbols periods. Figure 3b shows the phase estimation. The phase estimate (top, in radians) shows the phase estimate. The inset shows that the phase estimate has converged in less than 10 symbols. The Δ-phase (middle) shows Δϕ. The bottom plot shows the estimate of ω.

**Figure 4:**
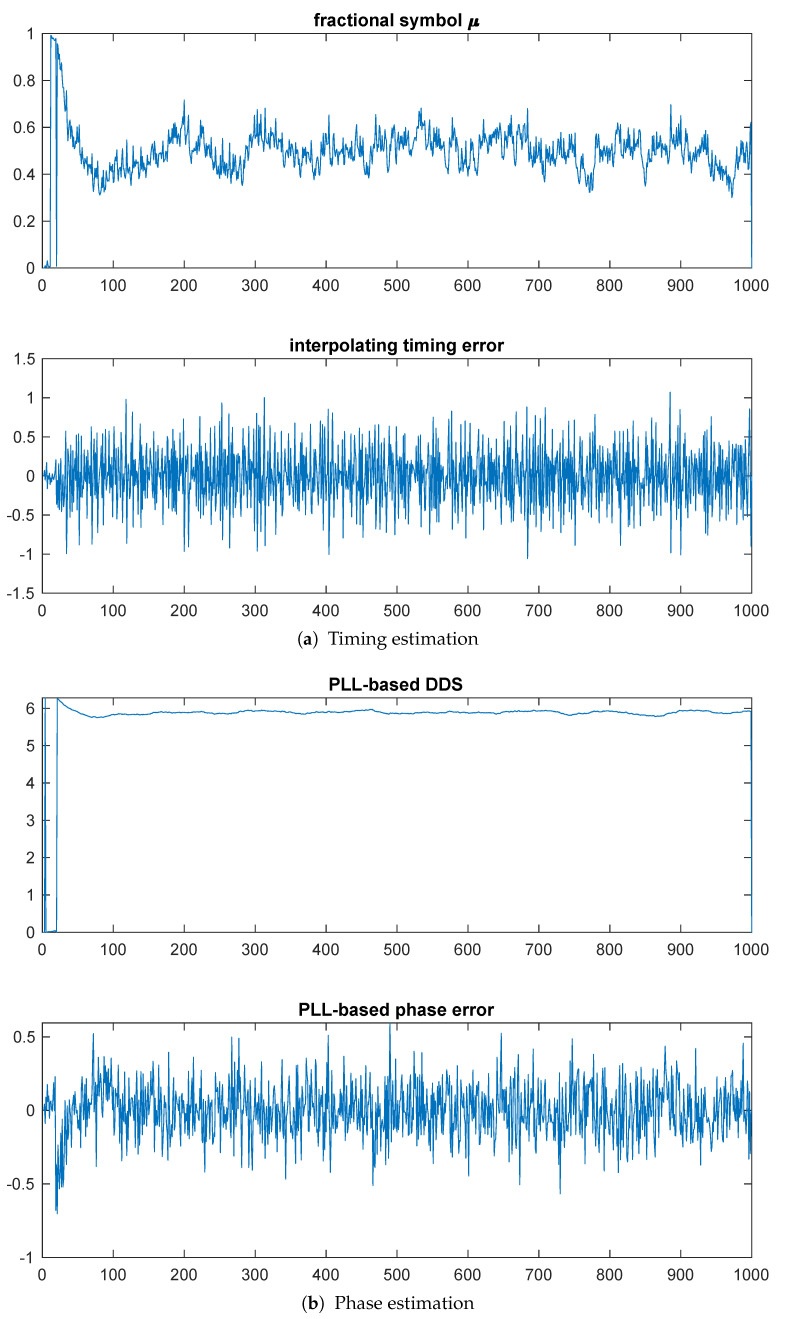
Example 1: PLL-based timing and phase estimation.

By comparison, Figure 4a shows the PLL-based estimates, with a filter time-bandwidth product $B_{N}T=0.01$. The top plot shows the fractional symbol μ, demonstrating convergence somewhere around 200 symbols. The bottom plot shows the phase converges in about 100 symbols.

As another method of comparing performance, the matched filter outputs were clustered (according to nearest signal constellation point) and the average variance of the clusters was computed. This variance was compared with the variance that would occur if the only source of noise were the AWGN. These variance results are shown in Table 1. In the column labeled “Noise Var”, the variance due to the AWGN at that SNR is shown. For example, in the first row where $E_{b}/N_{0}=10$ dB, the noise variance is 0.05. The column labeled “No Offsets” shows the result of estimating the variance of the clustered matched filter outputs when there are no phase or timing offsets. When $E_{b}/N_{0}=10$ dB, this is 0.05. This value should be close to the “Noise Var.” The columns labeled “$\delta_{H}$” and “Grad μ” indicate the settings for the parameters. “$\delta_{H}$” set to a value indicates that the Huber function was used. “Grad μ” set to a value indicates that gradient descent was used. The column labeled “Kurtosis” indicates the variance of the clustered matched filter outputs for the various kurtosis-based estimation algorithms after convergence. For example, the first row with $\delta_{H}=0.5$ shows that the variance is 0.061. This can be compared with the variance in the “No Offsets” column, indicating that using the estimate does, in fact, increase the variance of the matched filter outputs compared to not having to estimate the parameters at all. The column labeled “PLL-based“ shows the variance of the clustered matched filter outputs for the PLL-based estimators. For $E_{b}/N_{0}=10\mathrm{dB}$, this variance is 0.06. Finally, the column “dB(Kurt/PLL)” shows the comparison (in dB) between the kurtosis and PLL-based estimators, where negative numbers indicate superiority of kurtosis-based vs. PLL-based. For example, in the first row, kurtosis-based estimation is 0.06 dB worse (in variance) than PLL-based estimation.

As Table 1 shows that the variance performance between kurtosis-based and PLL-based estimation is generally quite close.

**Figure 5:**
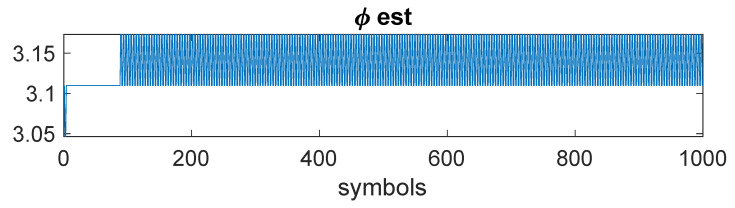
Example 2: Illustration of phase estimate jitter, $N_{\phi }=100$.

**Example** **2.** *This example shows behavior typical of the kurtosis-based phase estimate. In this case, $N_{\phi }=100$, and SNR = 24 dB. Figure 5 demonstrates an example of the phase estimate. After convergence, the estimator tends to jitter around the bottom of the kurtosis “bowl”, because the kurtosis is only estimated. The phase variance thus largely determined by the steps size, essentially $2\pi /N_{\phi }$.*

**Example** **3.** *QPSK, SNR = 10 dB, excess bandwidth = 0.2. $\omega_{off}=0.02$. Algorithm parameters: $N_{kurt}=20$, $N_{\tau }=60$, $N_{\phi }=40$. ω-filtering parameter α=0.95.*

**Figure 6:**
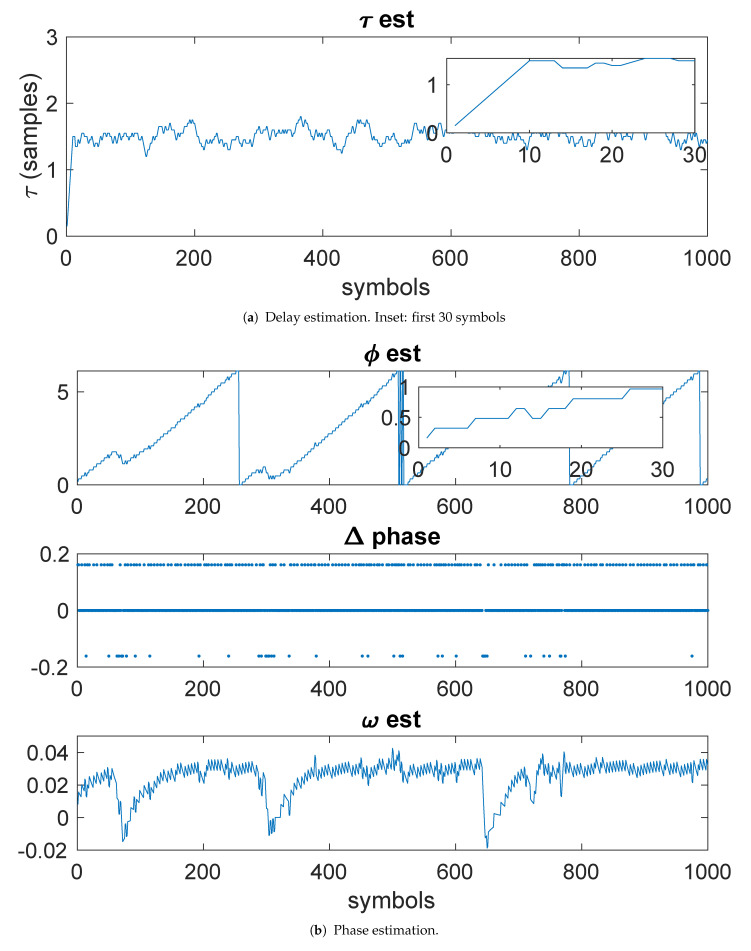
Example 3: Kurtosis-based estimates.

Figure 6a shows the results of the delay estimation. The inset in the figure shows the first 30 samples, illustrating that convergence occurred in less than 20 symbols. Figure 6b shows the phase estimation. The phase estimate (top, in radians) shows the phase estimate. The inset shows that the phase estimate converged in less than 10 symbols. The Δ-phase (middle) shows Δϕ. The bottom plot shows the estimate of ω.

**Figure 7:**
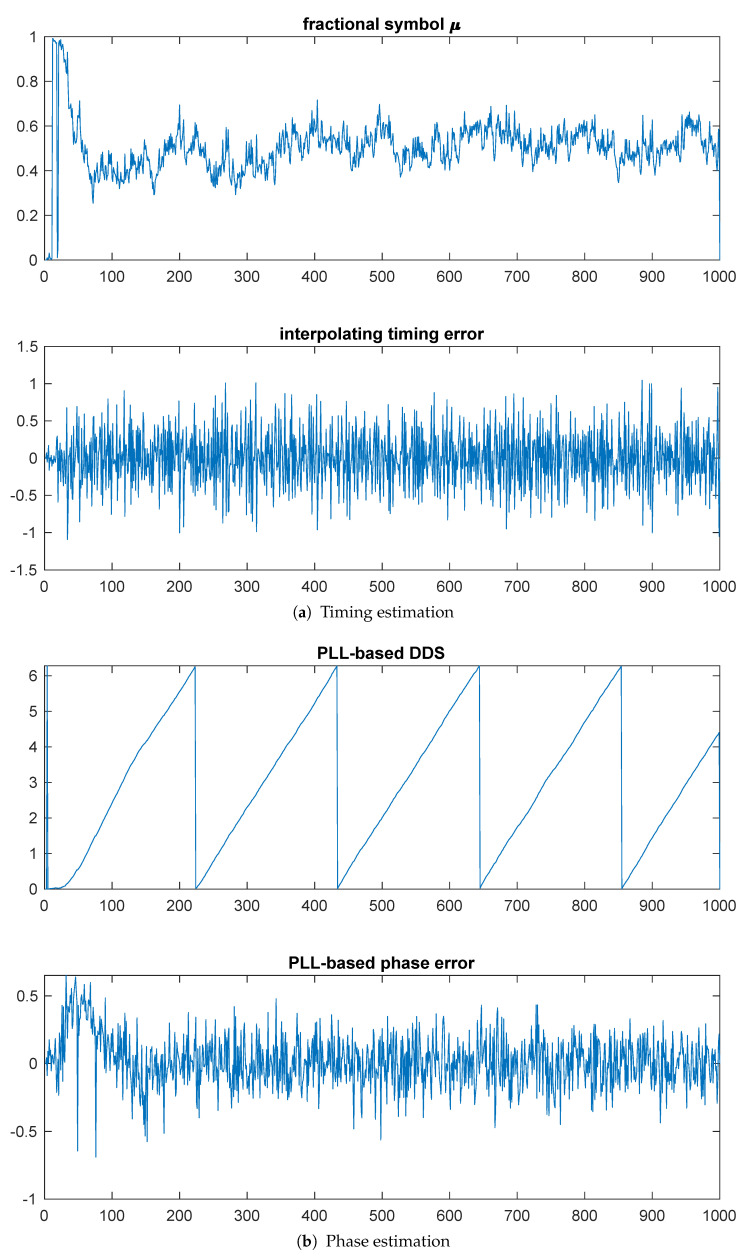
Example 3: PLL-based timing and phase estimation.

Figure 7 shows results for the PLL-based methods in this setting. While the phase tracking appears smoother in the PLL-based estimate, as the results for Example 3 in Table 1 show, there is less variance around the constellation points at the matched filter outputs for the kurtosis-based methods than the PLL-based methods when step descent is used. Interestingly, the gradient descent performs significantly worse than PLL-based estimation at all SNRs.

**Figure 8:**
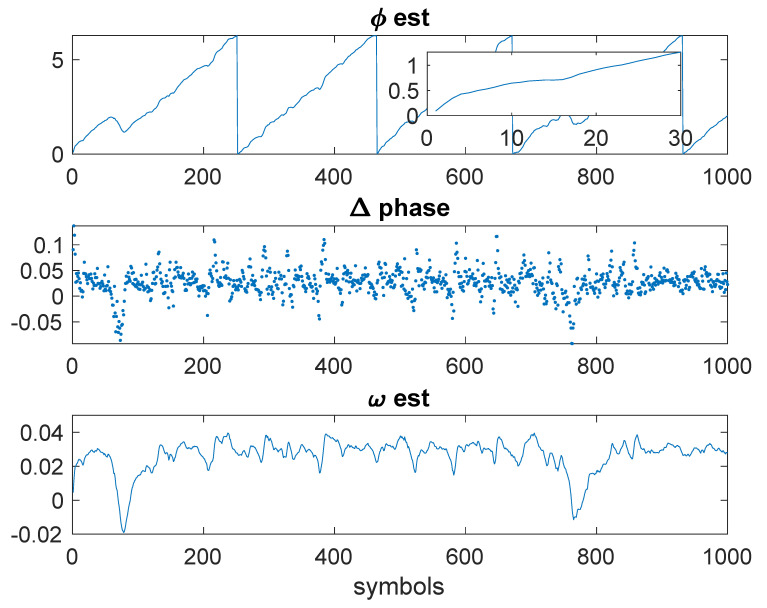
Example 3: Kurtosis-based estimates and phase estimation gradient descent.

Figure 8 shows results for the gradient descent estimation of the phase. The convergence time is about the same as the step descent, but there is higher variance on the estimate of ω.

## 9. Comparison with Modified Cramer–Rao Lower Bound

Evaluating the performance in terms of how the recovered signal points are clustered, as in Table 1, is a natural way to evaluate the performance of these estimation algorithms, since it demonstrates how all of the estimators work together to achieve what is desired in the receiver: good signal detection. Another way of evaluating performance of estimators is to compare the estimator variance against lower bounds such as the Cramer–Rao lower bound (CRLB) or modified CRLB. The modified CRLB provides a bound when the observed data depends on multiple parameters, but only one parameter at a time is estimated [22]. The modified CRLB is, in general, lower than the CRLB.

The modified CLRB for the estimate of ϕ is [22] (Equation (31))

$$MCRB\left(\phi \right)=\frac{B_{L}T_{s}}{E_{s}/N_{0}}$$

In [22], $B_{L}=1/\left(2LT_{s}\right)$, where $LT_{s}$ is the length of window over which the estimator operates. The modified CLRB for the estimate of τ is [22] (Equation (32)

(7) $$MCRB\left(\tau \right)=\frac{B_{L}T_{s}}{4\pi^{2}\xi }\frac{T_{s}^{2}}{E_{s}/N_{0}}$$

where

$$\xi =\frac{T_{s}^{2}\int_{-\infty }^{\infty }f^{2}{\left|P\left(f\right)\right|}^{2}\;df}{\int_{-\infty }^{\infty }{\left|P\left(f\right)\right|}^{2}\;df},$$

where P(f) is the Fourier transform of the pulse-shaping function p(t).

The kurtosis is computed over $N_{\mathrm{kurt}}$ symbols (matched filter outputs). The first matched filter output occurs at sample $QT_{s}/P$, with symbol outputs occuring every $T_{s}$ seconds thereafter. Thus, the duration over which a kurtosis is computed is $QT_{s}/P+\left(N_{\mathrm{kurt}}-1\right)T_{s}$. We take this as the value of $B_{L}T_{s}$ over which the estimate is computed. Since the estimator steps over several symbol times to converge, this does not apply initially but should be applicable after convergence.

**Figure 9:**
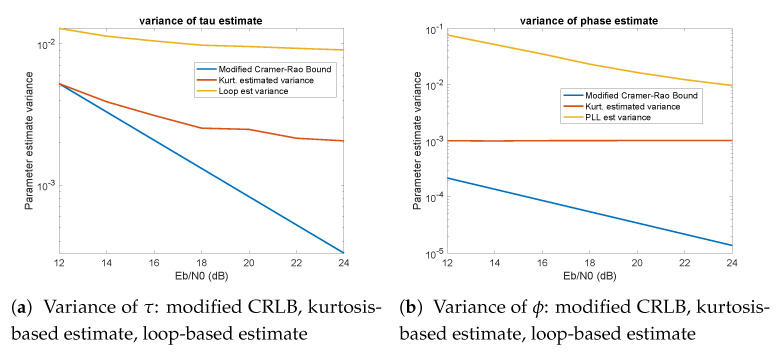
Variance of parameter estimates compared with modified CRLB.

Figure 9a shows the variance of estimate of τ using the kurtosis-based method (red) and the conventional (loop-based) method (yellow) compared with the modified CRLB (blue), as a function of $E_{b}/N_{0}$. The variance of the kurtosis-based method was obtained by computing the variance of $\hat{\tau }$ after convergence, such as seen in Figure 3a, averaged over 10 independent runs. The variance of the loop-estimated variance was computed as the variance of μ (the fractional timing offset in the synchronization algorithm [3]), averaged over 10 runs. The kurtosis-based method performs significantly better than the loop-based method and demonstrates variance decreases with SNR. However, it does not decrease as fast as the modified CRLB. Additionally, it appears that the kurtosis-based method meets the modified CRLB, which is unexpected. It may be that the value for $B_{L}T_{s}$ used to compute the bound in (7) is larger than it should be.

Figure 9b shows the variance of the estimate of ϕ using kurtosis (red) and PLL (yellow) compared with the modified CRLB (blue). The variance of the kurtosis-based method was obtained by computing the variance of $\hat{\phi }$ after convergence, such as seen in Figure 3b, averaged over 10 independent runs. The variance of the kurtosis method hardly changes with SNR (although it can be seen to vary slightly). The continued variation is due to the fixed step size, and the fact that the kurtosis estimate has some variance associated with it causing jitter in the indices. Depending on the value of $N_{\phi }$, this can achieve variances less than those of the PLL-based method. This suggests that a variable $N_{\phi }$ algorithm would be useful: use a larger step size to converge quickly, then reduce the step size to reduce the variance.

(These plots were performed using QPSK, Q=101, P=3, α=0.2, and $N_{\mathrm{kurt}}=40$ and using the Hubert function to estimate the kurtosis.)

## 10. Discussion and Conclusions

We demonstrated that kurtosis-based methods can be applied to symbol timing and phase estimation. These offer potential advantages such as being able to synchronize without knowledge of the signal constellation in a scale-invariant way and, generally, convergence as fast as or faster than PLL-based methods. The experiments showed that the variance of the matched filter outputs for kurtosis-based methods is about the same as or slightly better than PLL-based methods. The gradient descent phase estimation, however, does not generally perform as well as discrete minimization methods. We made the following observations:

- Using the Huber function instead of the full kurtosis reduces the variance of the estimators.
- For lower SNRs, the gradient-based method performs significantly worse than the discrete minimization.
- The gradient-based method has a higher complexity than the discrete minimization.
- Kurtosis-based methods converge more quickly than PLL-based methods. The number of steps is determined primarily by $N_{\tau }$, $N_{\phi }$, and $n_{\tau }$. (There is a secondary effect due to the bandwidth of the lowpass filter for ${\hat{\omega }}_{\mathrm{off}}$.)

The fast convergence time suggests that these kurtosis-based methods may be useful in situations where short packets are used, such as in the Internet of Things.

## Figures and Tables

**Table 1:** Cluster variances.

Example	$E_{b}/N_{0}$ (dB)	Noise Var	No Offsets	$\delta_{H}$	Grad μ	Kurtosis	PLL-Based	dB(Kurt/PLL)
1-9 1	10	0.05	0.05	0.5	—	0.061	0.06	0.06
				—	—	0.077		1.08
				0.5	0.01	0.054		−0.47
1-9	8	0.079	0.079	0.5	—	0.086	0.94	−0.38
				—	—	0.105		0.48
				0.5	0.01	0.128		−0.60
1-9	6	0.126	0.124	0.5	—	0.13	0.15	−0.47
				—	—	0.15		−0.016
				0.5	0.01	0.127		−0.64
1-9	4	0.2	0.19	0.5	—	0.19	0.22	−0.55
				—	—	0.22		0.056
				0.5	0.01	0.19		−0.62
1-9 2	10	0.05	0.05	0.5	—	0.057	0.6	−0.20
				—	—	0.058		−0.07
				0.5	0.2	0.21		5.4
1-9	8	0.079	0.079	0.5	—	0.085	0.093	−0.42
				—	—	0.088		−0.28
				—	—	0.23		3.91
1-9	6	0.079	0.079	0.5	—	0.13	0.15	−0.49
				—	—	0.15		0.16
				—	—	0.25		2.3
1-9	4	0.2	0.19	0.5	—	0.2	0.22	−0.51
				—	—	0.2		−0.49
				—	—	0.28		1.04

## Data Availability

Not Applicable.
